# Effect on Physical and Mechanical Properties of Conventional Glass Ionomer Luting Cements by Incorporation of All-Ceramic Additives: An In Vitro Study

**DOI:** 10.1155/2020/8896225

**Published:** 2020-09-30

**Authors:** Runki Saran, Nagraj P. Upadhya, Kishore Ginjupalli, Arul Amalan, Bharath Rao, Saurabh Kumar

**Affiliations:** ^1^Faculty of Dentistry, Melaka Manipal Medical College, Manipal Academy of Higher Education, Manipal 576104, India; ^2^Department of Dental Material, Manipal College of Dental Sciences, Manipal Academy of Higher Education, Manipal 576104, India; ^3^Department of Pedodontics & Preventive Dentistry, Manipal College of Dental Sciences, Manipal Academy of Higher Education, Manipal 576104, India

## Abstract

**Introduction:**

Glass ionomer cements (GICs) are commonly used for cementation of indirect restorations. However, one of their main drawbacks is their inferior mechanical properties.

**Aim:**

Compositional modification of conventional glass ionomer luting cements by incorporating two types of all-ceramic powders in varying concentrations and evaluation of their film thickness, setting time, and strength. *Material & Methods*. Experimental GICs were prepared by adding different concentrations of two all-ceramic powders (5%, 10, and 15% by weight) to the powder of the glass ionomer luting cements, and their setting time, film thickness, and compressive strength were determined. The Differential Scanning Calorimetry analysis was done to evaluate the kinetics of the setting reaction of the samples. The average particle size of the all-ceramic and glass ionomer powders was determined with the help of a particle size analyzer.

**Results:**

A significant increase in strength was observed in experimental GICs containing 10% all-ceramic powders. The experimental GICs with 5% all-ceramic powders showed no improvement in strength, whereas those containing 15% all-ceramic powders exhibited a marked decrease in strength. Setting time of all experimental GICs progressively increased with increasing concentration of all-ceramic powders. Film thickness of all experimental GICs was much higher than the recommended value for clinical application.

**Conclusion:**

10% concentration of the two all-ceramic powders can be regarded as the optimal concentration for enhancing the glass ionomer luting cements' strength. There was a significant increase in the setting time at this concentration, but it was within the limit specified by ISO 9917–1:2007 specifications for powder/liquid acid-base dental cements. Reducing the particle size of the all-ceramic powders may help in decreasing the film thickness, which is an essential parameter for the clinical performance of any luting cement.

## 1. Introduction

A significant increase in the number of partially edentulous individuals has been observed in the recent past. As a result, the demand for fixed partial dentures is on the rise [1]. Among the various dental cements available in the market, resin cements and glass ionomer cements (GIC) are the most frequently used for cementation of the fixed partial dentures. Resin cements are known for their excellent esthetics, strength, and resistance to dissolution in oral fluids. However, the main drawback of resin cement is that the placement of the cement is technique-sensitive. As these cements depend upon mechanical bonding to the tooth, the operator must be careful to follow all bonding steps like acid etching, application of bonding agent, etc., in proper order and with the recommended time for each step. Apart from this, the presence of residual monomer in the cement may raise some biocompatibility issues. The glass ionomer cement offers some distinct advantages over resin cement such as chemical bonding with the tooth structure, fluoride release, and recharging, which protects the tooth from occurrence of secondary caries and excellent biocompatibility with the pulp, to name a few. However, the relatively low strength of glass ionomer cements compared to resin cements is regarded as one of their most significant limitations [2].

The primary focus of our study was to enhance the mechanical properties of glass ionomer luting cement meant for cementation of permanent restorations. A cemented restoration's clinical performance depends upon two important factors, namely, structural integrity of the cement layer and quality of the adhesive bond. Previous studies with glass ionomer cements have revealed that the failure of cemented restoration occurs primarily due to fracture of the cement layer, indicating the inherent lack of strength of the cement [3].

Several recent studies have found that a positive correlation exists between the inherent strength and the adhesive strength of the cement. Most of the luting glass ionomer cements possess a compressive strength ranging between 80 MPa and 120 MPa, which compromises their adhesive property. Research suggests that an improvement in compressive strength may enhance their adhesive strength and result in better clinical performance [4]. Strength can be improved by modifying the matrix composition or by the addition of certain external additives that act as reinforcing agents.

Over the years, numerous modifications have been made to the liquid and powder components of glass ionomer cements to enhance their mechanical properties. Most of these studies have been carried out on glass ionomer cements, which are meant for restoration. Bioactive glass (BAG) particles in different concentrations have been added to GICs to enhance their mechanical properties [5, 6]. The increase in BAG content resulted in better antibacterial property but decreased the strength of GIC. In a recent study, the addition of Al^3+^ to the BAG-GIC combinations was shown to improve the strength but reduce the bioactivity [7]. A significant decrease in strength, working time, and initial setting time was observed in restorative GICs containing different concentrations of barium sulfate and ytterbium fluoride [8]. The addition of GeO_2_, ZrO_2_, and Na_2_O to GIC bone cements did not produce any significant effect on their strength but improved the handling characteristics significantly [9–11]. Several studies have evaluated the effect of incorporating silica on the mechanical properties of restorative GICs [12, 13]. An experimental, restorative GIC containing nano-hydroxyapatite silica particles exhibited significantly higher strength and hardness [14]. Incorporation of niobium pentaoxide to luting GICs was reported to decrease their compressive strength significantly [15]. However, in recent years, it has been demonstrated that 5% niobium pentoxide can be added to improve the radiopacity of GIC without affecting its strength [16].

The effect of incorporating different reactive glass fibers on the strength of Glass ionomer restorative material has also been evaluated. Reinforcement of a glass ionomer cement with 20 vol% short glass fibers, (430 *μ*m) was shown to result in higher fracture toughness [17]. Addition of 60 wt% of short glass fibers was also reported to increase the diametral tensile strength and flexural strength of GICs [18]. Similarly, diametral tensile strength, flexural strength, flexural modulus, and fracture toughness was found to be higher in glass ionomer restorative cements containing 3 wt% and 5 wt% of short glass fiber [19]. GICs containing hollow discontinuous glass fibers exhibited higher flexural strength and fracture toughness, but the compressive strength remained unchanged [20].

Titanium dioxide nanotubes in different concentrations have been incorporated into glass ionomer restorative cements to enhance their physical, chemical, and biological properties [21]. Of late, silver nanoparticles and fluorinated graphene have been incorporated in experimental GICs, which significantly enhanced their mechanical as well as antibacterial properties [22, 23].

This study was directed towards modifying the composition of conventional glass ionomer luting cements by adding all-ceramic powders to improve their strength. The all-ceramic powders were chosen as additives in our study as they are known to be biocompatible and insoluble in oral fluids, have chemical compatibility with the glass powder, and possess high hardness and compressive strength [24]. Therefore, they may act as reinforcing agents and improve the mechanical properties of the glass ionomer cement.

The main objective of our study was to evaluate the effect of varying concentrations of all-ceramic powders on the strength, film thickness, and setting time of conventional glass ionomer luting cements.

The null hypothesis was that the addition of all-ceramic powders to conventional glass ionomer luting cements will not affect their strength, film thickness, and setting time.

## 2. Material and Methods

Ketac™ Cem Radiopaque Permanent Glass Ionomer Luting Cement, 3M ESPE United States, and GC Corporation Gold Label Type 1 Glass Ionomer Luting and Lining Cement, GC corporation, Tokyo, Japan, were the two conventional glass ionomer cements used. Two varieties of all-ceramic powder, IPS Empress 2 Incisal, Ivoclar Vivadent, Liechtenstein and IPS Empress 2 Dentin, Ivoclar Vivadent, Liechtenstein, were used as additives.

For the preparation of the experimental glass ionomer cements, different concentrations of the all-ceramic powders (5%, 10%, and 15% by weight) were thoroughly blended with the glass ionomer powder to obtain a homogeneous distribution. Cements without incorporation of all-ceramic additives in the glass ionomer powder were used as control. The prepared experimental glass ionomer cements were categorized into two groups, as shown in Table 1.

The experimental glass ionomer powders were mixed with the liquid according to the recommended powder :  liquid ratio. Based on the results of pilot work and similar studies published [25, 26], a sample size of 6 for each experimental glass ionomer cement and control in a group was considered for measuring the properties (i.e. *n* = 6). The compressive strength, film thickness, and initial and final setting time were measured as per the ISO 9917–1:2007 specifications for powder/liquid acid-base dental cements intended for permanent cementation. While performing the tests, the operator was blinded to eliminate any operator bias. This was done by coding the samples so that the operator could not know the actual experimental group to which they belonged.

### 2.1. Compressive Strength Measurement

For measuring compressive strength, cylindrical samples were prepared by placing freshly mixed cement into a split mold with internal dimensions of 6 mm height and 4 mm diameter. Samples were taken out from the mold after one hour and kept in distilled water at 37°C and 50% relative humidity for the next twenty-three hours. Samples were then removed from distilled water and dried with blotting paper. The compressive strength of the samples was measured using a universal testing machine (Instron3366, Buckinghamshire, UK) at a crosshead speed of 0.5 mm/min [27]. Six specimens were made for each experimental glass ionomer cement and control in a group, and their mean compressive strength was recorded.

### 2.2. Film Thickness Measurement

To measure the film thickness, two glass plates that were optically flat, square in shape, 5 mm thick and had 200 mm^2^ contact surface area were used [28, 29]. The glass plates were stacked, and their combined thickness (T1) was measured using a dial gauge. Film thickness was determined by keeping 0.1 ml of the freshly mixed cement in the middle of the lower plate, covering it with the upper plate, and applying a load of 150 ± 2 N for 10 min. The thickness of glass plates, together with the cement mix between them, was then measured (T2). The film thickness was calculated using the following formula:

(1) $$\begin{array}{c} \text{Film thickness}=\left(\text{T}2-\text{T}1\right)\;\mu . \end{array}$$

The test was repeated six times each for the control and experimental GICs, and the mean value was noted.

### 2.3. Setting Time Measurement

Initial and final setting time were measured with a small indenter (28 g) and a large indenter (500 g), respectively. Freshly mixed cement was placed in a metal mold having a 10 mm diameter and 2 mm thickness. Ninety seconds after the end of mixing, the small indenter was carefully lowered vertically onto the cement's surface and allowed to remain there for 5 seconds. A trial run was carried out to determine the approximate setting time, repeating the indentations at 30 seconds intervals until the needle failed to make a complete circular indentation in the cement when viewed using 2X magnification. The process was repeated, starting the indentation at 30 seconds before the approximate setting time, making indentations at 10 seconds intervals. This gave the initial setting time. Then the large indenter was lowered vertically onto the cement's surface and allowed to remain there for 5 seconds. The indentations were repeated at 30 seconds intervals until the needle failed to make a complete circular indentation in the cement when seen using 2X magnification. The final setting time was recorded as the time elapsed from the end of mixing to the time when the needle failed to make a complete circular indentation with a larger indenter in the cement [30]. The test was repeated six times each for the control and experimental GICs, and the mean value was noted.

### 2.4. Differential Scanning Calorimetry (DSC)

DSC-60 (Shimadzu Corp, Japan) was used for differential scanning calorimetry analysis of the set cement. The samples were subjected to a dynamic temperature program and heated from 0°C to 200°C at a rate of 5°C per minute. DSC operating software was used for qualitative as well as quantitative assessment of the thermograms.

### 2.5. Particle Size Analysis

The particle size of the GICs used as control and the all-ceramic powders were determined using CILAS 1064 Particle Size Analyser.

All data was analyzed using one-way ANOVA and Tukey post hoc analysis at a confidence interval of 95% (*P* < 0.05).

## 3. Result

### 3.1. Compressive Strength

**Figure 1:**
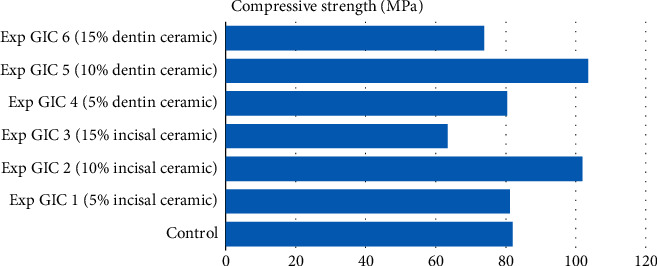
Compressive strength of samples in Group 1.

In Group 1, experimental GIC 2 and experimental GIC 5 containing 10% incisal ceramic powder and 10% dentin ceramic powder showed significantly higher compressive strength than the control (see Table 2). Experimental GIC 1 with 5% incisal ceramic powder and Experimental GIC 4 with 5% dentin ceramic powder had no improvement in strength than the control. Moreover, in both Experimental GIC 3 and 6 containing 15% incisal ceramic and 15% dentin ceramic powder, respectively, a marked decrease in strength, though statistically not significant, was observed (see Figure 1).

**Figure 2:**
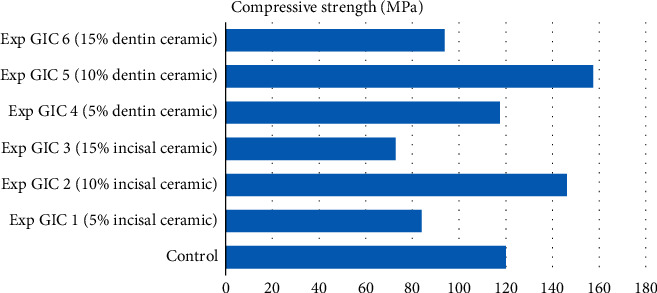
Compressive strength of samples in Group 2.

The experimental glass ionomer cements of Group 2 exhibited a similar trend (see Table 2 and Figure 2). The compressive strengths of experimental GIC 2 and experimental GIC 5 containing 10% incisal ceramic powder and 10% dentin ceramic powder, respectively, were significantly higher than those of the control. Experimental GIC 1 with 5% incisal ceramic powder and Experimental GIC 4 with 5% dentin ceramic powder showed no improvement in strength than the control. Although the compressive strength of both Experimental GIC 3 and 6 containing 15% incisal ceramic and 15% dentin ceramic powder was lower than that of the control, the difference was statistically significant only in experimental GIC 6.

### 3.2. Film Thickness

**Figure 3:**
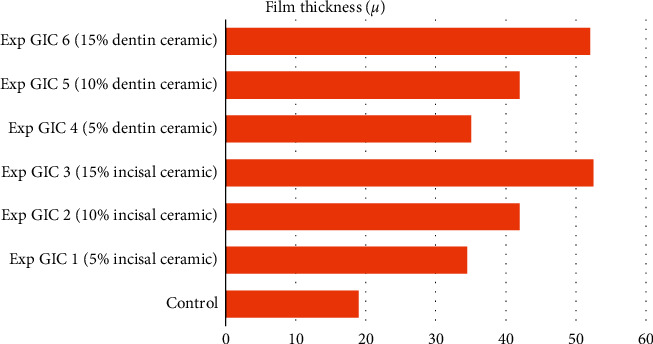
Film thickness of samples in Group 1.

**Figure 4:**
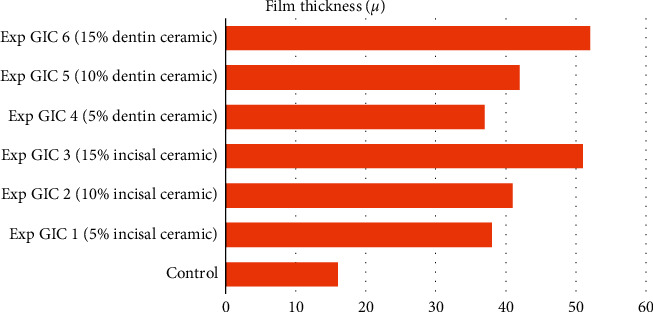
Film thickness of samples in Group 2.

The film thickness of the control in group 1 was 20 *μ*, whereas that of the control in group 2 was 17 *μ*. In both Figures 1 and 2, the film thickness of all the experimental GICs was found to be significantly higher than that of the control (see Table 3). A progressive increase in film thickness was observed with increasing concentration of the incorporated ceramic powders (see Figures 3 and 4).

### 3.3. Initial and Final Setting Time

**Figure 5:**
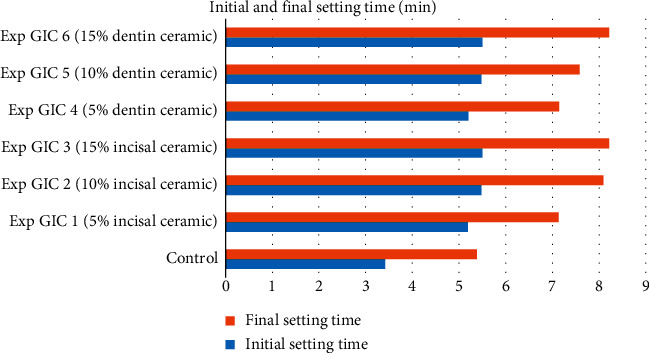
Setting time of samples in Group 1.

**Figure 6:**
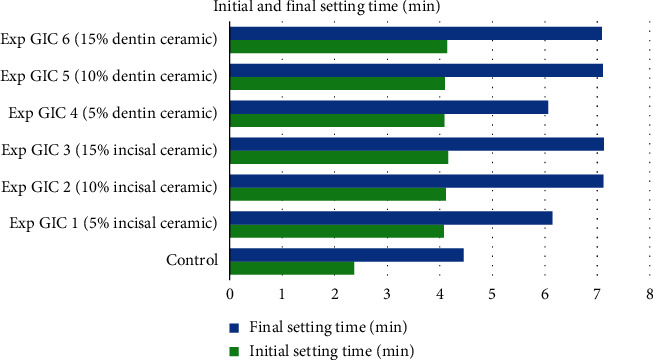
Setting time of samples in Group 2.

All the experimental GICs in groups 1 and 2 had significantly longer initial and final setting time compared with the controls (see Table 3). Moreover, it was observed that an increase in the concentration of the ceramic powders resulted in longer initial and final setting time of the experimental GICs (see Figures 5 and 6).

### 3.4. Differential Scanning Calorimetry (DSC) Analysis

**Figure 7:**
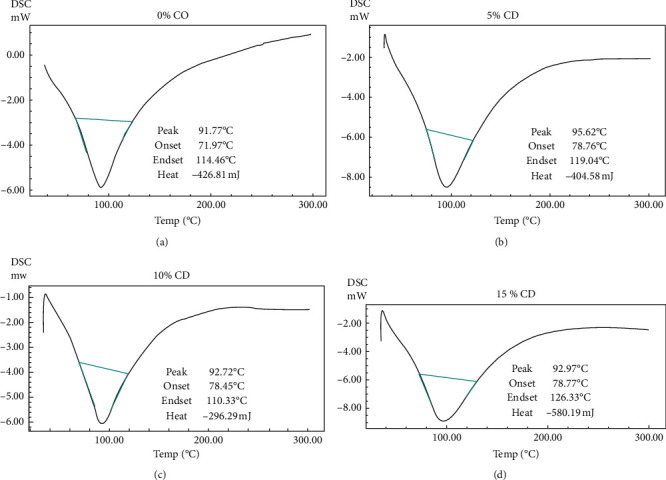
(a) Differential Scanning Calorimetry (DSC) analysis of Control of group 2. (b) Differential Scanning Calorimetry (DSC) analysis of experimental GIC 4. of group 2 (with 5% dentin ceramic powder). (c) Differential Scanning Calorimetry (DSC) analysis of experimental GIC 5. of group 2 (with 10% dentin ceramic powder). (d) Differential Scanning Calorimetry (DSC) analysis of experimental GIC 6 of group 2 (with 15% dentin ceramic powder).

The enthalpy would have got altered if the sample underwent a physical or chemical change. The differential scanning calorimetry analysis in our study showed greater enthalpy changes in the experimental glass ionomer cements compared to the control, which is indicative of delayed setting reaction (see Figures 7(a)–7(d)).

### 3.5. Particle Size Analysis

The glass ionomer cements used as control in the study had an average particle size of 18 *μ*, whereas the average particle size of the two of all-ceramic powders was 30 *μ*.

## 4. Discussion

All experimental GICs demonstrated compressive strengths well above the ISO requirement for dental cements intended for permanent cementation of no less than 50 MPa. A significant increase in strength was exhibited by experimental GICs containing 10% all-ceramic additives in both groups compared to their controls.

The cement's compressive strength depends upon the matrix formation, and concentration of the ceramic powder was added. In order to improve the strength of the glass ionomer cement, the added ceramic powder particles must act as inert reinforcing fillers and allow adequate matrix formation during setting of the cement. In our study, the experimental GICs containing 10% concentration of the all-ceramic additives showed significantly higher strength. Therefore, 10% concentration of the ceramic additives can be considered the optimal concentration required for the reinforcement. The experimental GICs containing 5% concentration of the two all-ceramic additives exhibited no significant improvement in strength. This could be due to the fact that, at such low concentration, the amount of ceramic additives is not enough to provide any reinforcement. Compressive strength of experimental GICs with 15% all-ceramic powders was much lower than that of control, and this could be because of inadequate matrix formation. A constant powder : liquid ratio was followed for mixing all the experimental GICs; this implies that when high concentrations of ceramic powders are added, a proportionate decrease in the glass ionomer powder has to be made. Thus, in experimental GICs containing 15% ceramic powder, lesser amount of glass powder is present to react and crosslink with the polyacid chains. Hence the amount of matrix formed is insufficient to improve the strength [31].

The setting reaction of glass ionomer cements involves an acid-base reaction between the glass powder and the liquid containing primarily an aqueous solution of polyacrylic acid [32, 33]. The first step of the reaction involves the dissolution of the glass particle's surface by the polyacrylic acid, followed by the release of metal ions like Al^3+^ and Ca^2+^ ions from the surface. The cement's initial setting (gelation) involves crosslinking of calcium ions with the carboxylate groups of the polyacid, resulting in the formation of calcium polyacrylate matrix. The cement's final setting, known as “maturation,” takes place with the crosslinking of the aluminum ions with the carboxylate groups of the polyacid. The aluminum polyacrylate matrix, thus formed, is responsible for the strength of the set cement. The cement takes about 24 hours for maturation, and over time, there is a gradual increase in strength for months [34].

In our study, all the experimental GICs in groups 1 and 2 had significantly longer initial and final setting time compared with the controls. This could be due to hindrance in the crosslinking of the calcium with the polyacid chain, which may have delayed the initial matrix formation or gelation. This finding was confirmed by the differential scanning calorimetry (DSC) analysis of the set cements (Figures 7(a)–7(d)). The experimental GICs exhibited greater enthalpy changes, which suggests delayed setting reaction [35, 36]. These findings indicate that the incorporation of ceramic powder acts as a physical obstacle and interferes with the setting reaction of cement, by inhibiting the crosslinking between polyacid chains and calcium ions. At this concentration, there was a noticeable increase in the initial and final setting time. Still, it was within the limit given by ISO 9917–1:2007 specifications, according to which, the net setting time should be within 1.5 to 8 minutes. A slight increase in initial setting time is beneficial for the dentist as it offers more time for manipulation of cement, its application on the prosthesis, and proper seating of the prosthesis on the prepared tooth.

The film thickness of luting cement is one of the essential rheological properties that enable the restorations to be seated properly on the prepared teeth [37]. According to ISO 9917–1:2007 specifications, the film thickness of luting cements should not exceed 25 *μ*. All the experimental GICs in our study had film thickness much higher than 25 *μ*. The film thickness of the experimental GICs in both groups was found to increase progressively with increase in the concentration of the two ceramic powders. Particle size analysis showed that the particle size of the two type 1 glass ionomer cements was around 18 *μ*, whereas the same for the two all-ceramic powders was around 35 *μ*. Such a significant difference in the particles' size made it difficult to blend the ceramic powders with glass ionomer powders satisfactorily, resulting in high film thickness. A film thickness of more than 25 *μ* is clinically unacceptable as it may affect the prosthesis's proper seating over the prepared tooth.

Our study's null hypothesis was that addition of all-ceramic powders to conventional glass ionomer luting cements will not affect their strength, film thickness, and setting time. The results obtained from our study showed that addition of all-ceramic powders to conventional glass ionomer luting cements did affect their strength, film thickness, and setting time. Hence, the null hypothesis was rejected.

## 5. Conclusion

From our study, we can conclude that the addition of 10% concentration of the two all-ceramic powders successfully increased the strength of both glass ionomer cements used in the study. However, the film thickness of these cements was much higher than the specifications given by ISO. Further research needs to be done to reduce the film thickness of the experimental GICs. One way to obtain a lower film thickness could be by reducing the particle size of the ceramic powders by procedures such as ball milling so that it is within the same range as that of glass ionomer powders. The effect of these all-ceramics additives on fluoride release, solubility, biocompatibility, and esthetics of glass ionomer cements also needs to be assessed in future studies.

## Figures and Tables

**Table 1:** Two groups of control and prepared experimental glass ionomer cements.

Group 1	Group 2
Control–Ketac™ cem with 0% ceramic powder	Control-GC corporation gold label type 1 glass ionomer luting and lining cement with 0% ceramic powder
Experimental GIC 1 Ketac™ cem with 5% IPS empress 2 incisal ceramic powder	Experimental GIC 1-GC corporation gold label type 1 glass ionomer luting and lining cement with 5% IPS empress 2 incisal ceramic powder
Experimental GIC 2 Ketac™ cem with 10% IPS empress 2 incisal ceramic powder	Experimental GIC 2-GC corporation gold label type 1 glass ionomer luting and lining cement with 10% IPS empress 2 incisal ceramic powder
Experimental GIC 3 ketac cem with 15% IPS empress 2 incisal ceramic powder	Experimental GIC 3-GC corporation gold label type 1 glass ionomer luting and lining cement with 15% IPS empress 2 incisal ceramic powder
Experimental GIC 4 Ketac™ cem with 5% IPS empress 2 dentin ceramic powder	Experimental GIC 4-GC corporation gold label type 1 glass ionomer luting and lining cement with 5% IPS empress 2 dentin ceramic powder
Experimental GIC 5 Ketac™ cem with 10% IPS empress 2 dentin ceramic powder	Experimental GIC 5-GC corporation gold label type 1 glass ionomer luting and lining cement with 10% IPS empress 2 dentin ceramic powder
Experimental GIC 6 Ketac™ cem with 15% IPS empress 2 dentin ceramic powder	Experimental GIC 6-GC corporation gold label type 1 glass ionomer luting and lining cement with 15% IPS empress 2 dentin ceramic powder

**Table 2:** Compressive strength of samples in Group 1 and 2.

Group 1		Group 2	
	Compressive strength (MPa)		Compressive strength (MPa)
Control	81.92	Control	120.16
Experimental GIC 1	81.13	Experimental GIC 1	83.93
Experimental GIC 2	101.85^∗^	Experimental GIC 2	146.19^∗^
Experimental GIC 3	63.37	Experimental GIC 3	72.91
Experimental GIC 4	80.46	Experimental GIC 4	117.56
Experimental GIC 5	103.48^∗^	Experimental GIC 5	157.39^∗^
Experimental GIC 6	73.76	Experimental GIC 6	93.87^∗^

^∗^The symbol indicates that there was significant difference between the experimental GIC and control within the group (*p* < 0.05), *n* = 6.

**Table 3:** Film thickness and setting time of samples in Groups 1 and 2.

Group 1				Group 2			
	Film thickness (*μ*)	Initial setting time (min)	Final setting time (min)		Film thickness (*μ*)	Initial setting time (min)	Final setting time (min)
Control	19	3.42	5.38	Control	16	2.37	4.45
Experimental GIC 1	34.5^∗^	5.19^∗^	7.13^∗^	Experimental GIC 1	38^∗^	4.08^∗^	6.14^∗^
Experimental GIC 2	42^∗^	5.48^∗^	8.09^∗^	Experimental GIC 2	41^∗^	4.11^∗^	7.11^∗^
Experimental GIC 3	52.5^∗^	5.50^∗^	8.21^∗^	Experimental GIC 3	51^∗^	4.16^∗^	7.12^∗^
Experimental GIC 4	35^∗^	5.2^∗^	7.14^∗^	Experimental GIC 4	37^∗^	4.09^∗^	6.06^∗^
Experimental GIC 5	42^∗^	5.48^∗^	7.58^∗^	Experimental GIC 5	42^∗^	4.10^∗^	7.10^∗^
Experimental GIC 6	52^∗^	5.50^∗^	8.21^∗^	Experimental GIC 6	52^∗^	4.14^∗^	7.09^∗^

^∗^The symbol indicated that there was significant difference between the experimental GIC and control within the group, (*p* < 0.05), *n* = 6.

## Data Availability

The data used to support the findings of this study are available from the corresponding author upon request.
